# The molecular phylogeny of eph receptors and ephrin ligands

**DOI:** 10.1186/1471-2121-9-27

**Published:** 2008-05-21

**Authors:** Dan O Mellott, Robert D Burke

**Affiliations:** 1 Department of Biochemistry and Microbiology, University of Victoria, Victoria, Canada; 2 Department of Biology, University of Victoria, Victoria, Canada

## Abstract

**Background:**

The tissue distributions and functions of Eph receptors and their ephrin ligands have been well studied, however less is known about their evolutionary history. We have undertaken a phylogenetic analysis of Eph receptors and ephrins from a number of invertebrate and vertebrate species.

**Results:**

Our findings indicate that Eph receptors form three major clades: one comprised of non-chordate and cephalochordate Eph receptors, a second comprised of urochordate Eph receptors, and a third comprised of vertebrate Eph receptors. Ephrins, on the other hand, fall into either a clade made up of the non-chordate and cephalochordate ephrins plus the urochordate and vertebrate ephrin-Bs or a clade made up of the urochordate and vertebrate ephrin-As.

**Conclusion:**

We have concluded that Eph receptors and ephrins diverged into A and B-types at different points in their evolutionary history, such that primitive chordates likely possessed an ancestral ephrin-A and an ancestral ephrin-B, but only a single Eph receptor. Furthermore, ephrin-As appear to have arisen in the common ancestor of urochordates and vertebrates, whereas ephrin-Bs have a more ancient bilaterian origin. Ancestral ephrin-B-like ligands had transmembrane domains; as GPI anchors appear to have arisen or been lost at least 3 times.

## Background

The Eph family represents the largest subgroup of receptor tyrosine kinases, with most vertebrate genomes having 14 members [1]. Eph receptors can be divided into two classes based on sequence similarity and ligand binding affinity [2,3]. A-type Eph receptors (EphA1–8,10) bind promiscuously to glycosylphosphatidylinositol (GPI) – linked ephrin-A ligands (ephrin-A1–6), whereas B-type Eph receptors (EphB1–4, 6) bind promiscuously to transmembrane ephrin-B ligands (ephrin-B1–3). The only known exceptions to this are EphA4, which can bind ephrin-B2 and 3 in addition to the ephrin-As [3], and EphB2, which can bind ephrin-A5 in addition to the ephrin-Bs [4].

Eph receptors and ephrin ligands classically function in cell-cell repulsion events. For example, retinal ganglion cell (RGC) axons expressing high levels of Eph receptors are excluded from ephrin-rich regions in the optic tectum and instead map to points where ligand expression is low [5,6]. Eph receptors and ephrins similarly show complementary distributions in the developing hindbrain, where their interactions function to keep different cell populations separate and thus establish segment boundaries [7]. Eph receptors are also expressed by the neural crest (NC) and mediate the exclusion of these cells from territories where complementary ephrin ligands are expressed, thus restricting their migration to stereotypical pathways [8,9].

Eph receptors and ephrins are found throughout the metazoans and the phylogeny of these genes in vertebrates is well established [10,11]. However, the relationship of invertebrate Eph receptors and ephrins to their vertebrate counterparts is not as clear [12]. A number of studies have constructed phylogenies using vertebrate and ecdysozoan (e.g. nematode and insect) sequences, generally resulting in trees with three major clades: one comprised of ecdysozoan Eph receptors/ephrins, a second comprised of vertebrate EphAs/ephrin-As, and a third comprised of vertebrate EphBs/ephrin-Bs [11,13,14]. However, this approach is unsatisfactory because of the large evolutionary gaps between the protostomes and the vertebrates. The increasing availability of genomic data from deuterostomes such as echinoderms, urochordates and cephalochordates presents an opportunity to better resolve the question of how Eph receptors and ephrins diversified.

## Results

The amino acid sequences of the full-length Eph receptors could be readily aligned with the ephrin binding domains and the tyrosine kinase domains being most highly conserved and acting as anchors (see Additional file 1). Gaps were introduced principally by inclusion of the human EphA6 and *C. intestinalis *Eph sequences. The full-length Eph sequences ranged from 10% to 68% identity, whereas the sequences between the ephrin binding domain and the kinase domain ranged from 30 to 60% identity. Trees constructed from full-length sequences, sequences between the Ephrin binding and kinase domains, or from the kinase domain alone all had similar topology. The full-length Ephrin amino acid sequences will also readily align with ClustalW centered on the conserved Ephrin domain (see Additional file 2). The amino terminal sequence of *N. vectensis *and the amino and carboxyl sequences of *D. melanogaster *sequence are unique and were removed from the analysis. Gaps were introduced to accommodate the *D. melanogaster *and *C. elegans *sequences. The overall identity within the Ephrin sequences was from 10 to 60%, whereas within the Ephrin domain identities ranged from 30 to 60%. Several of the sequences used are predictions made from genomic data and introns exon boundary predictions may be incorrect.

**Figure 1:**
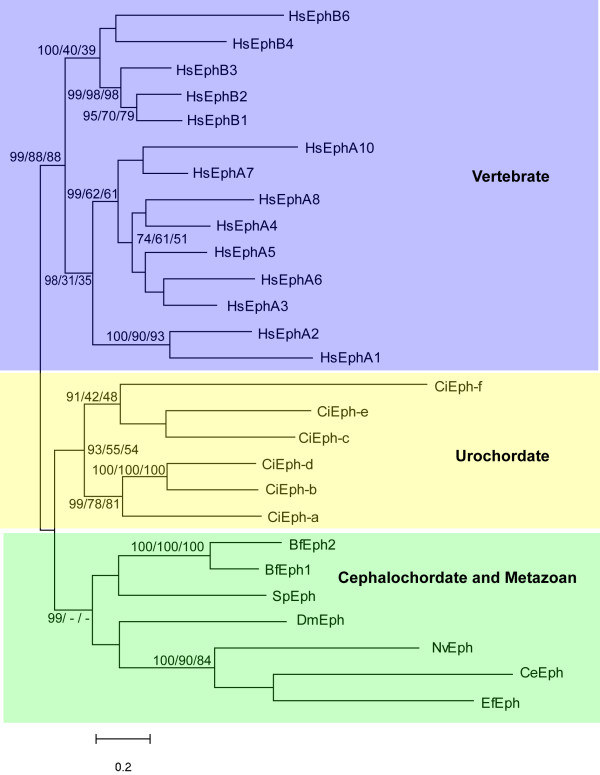
**Unrooted phylogenetic tree for Eph receptors constructed with the Maximum Likelihood method.** Numbers represent percentage bootstrap values for 1000 replicates (Maximum likelihood/Neighbour Joining/Minimum Evolution); unlabelled branches or/- indicates a value less that 50%. Three major clades are present: Vertebrate, Urochordate, and a group containing Cephalochordates, Echinoderms, Ecdysozoans, Coelenterate and Sponges. Using the ecdysozoan branch to root the tree does not change the composition of the major groups. Bf, *Brachiostoma floridae*; Ce, *Caenorhabditis elegans*; Ci, *Ciona intestinalis*; Dm, *Drosophila melanogaster*; Ef, *Ephydatia fluviatilis*; Hs, *Homo sapiens*; Nv, *Nematostela vectensis*; Sp, *Strongylocentrotus purpuratus*.

Our phylogenetic tree for invertebrate and vertebrate Eph receptors is comprised of a cephalochordate plus non-chordate clade, a urochordate clade, and a vertebrate clade (Fig. 1). There is weak support for the Urochordate and Cephalochordate/Metazoan grouping, however independent diversification of urochordate, cephalochordate, and ecdysozoan clades is well supported. With this larger data set, there is a distinct vertebrate Eph clade divided into a EphA and EphB groups. Within the EphA clade, there is strong bootstrap support for a subgroup comprised of EphA3–6,8 and another comprised of EphA7 and 10. Similarly, within the EphB clade, there is a distinct group comprised of Eph4,6 and another comprised of Eph1–3.

**Figure 2:**
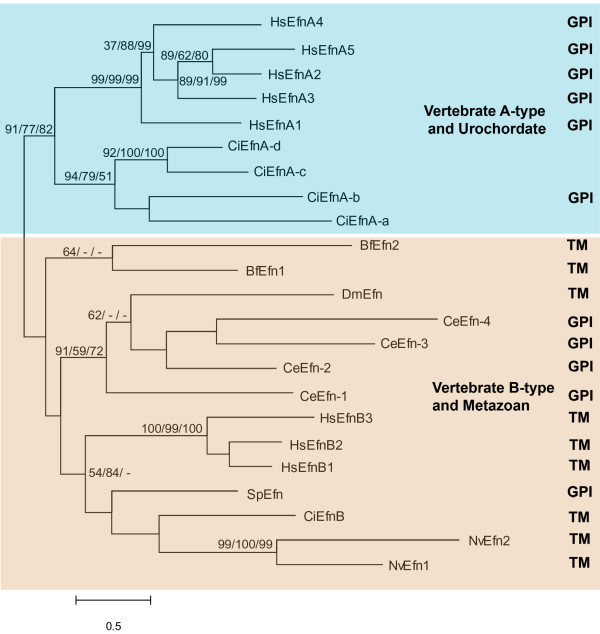
**Phylogenetic tree for ephrin ligands constructed with Maximum Likelihood method.** Numbers represent percentage bootstrap values for 1000 replicates (Maximum likelihood/Neighbour Joining/Minimum Evolution); unlabelled branches or/- indicates a value less that 50%. Two major clades are present: Vertebrate A-type and Urochordate ephrins and a group containing Vertebrate B-type, Cephalochordate, Ecdysozoan, Echinoderm, Coelenterate, Sponge and one Urochordate ephrin. Using the ecdysozoan branch to root the tree does not change the composition of the major groups. Efn, ephrin; Bf, *Brachiostoma floridae*; Ce, *Caenorhabditis. elegans*; Ci, *Ciona intestinalis*; Dm, *Drosophila melanogaster*; Hs, *Homo sapiens*; Nv, *Nematostella vectensis*; Sp, *Strongylocentrotus purpuratus*. Results of predictions for the type of membrane anchorage are aligned with individual branches; TM, transmembrane domains; GPI, glycosylphosphatidylinositol modification. Where no prediction is reported the protein prediction contains neither.

Our phylogenetic tree for ephrin ligands can be broadly divided into two major clades (Fig. 2). One diverse group includes coelenterate, ecdysozoan, echinoderm, cephalochordate, urochordate and vertebrate B ephrins. Within this group there is evidence for independent diversification of cephalochordate, ecdysozoan and deuterostome ephrin ligands. The second major ephrin clade is comprised of a urochordate ephrin-A subclade and a vertebrate ephrin-A subclade. There is good support for this being a monophyletic group in which there have been independent diversifications.

## Discussion

The clearest implication from our phylogenetic analysis is that Eph receptors and their ephrin ligands diversified at different times in evolutionary history. Vertebrate EphAs and EphBs do not have invertebrate orthologues, whereas vertebrate ephrin-As and ephrin-Bs have urochordate counterparts. This suggests that the diversification of Eph receptors followed the diversification of ephrins and that the common ancestor of urochordates and vertebrates likely had a single receptor for the two different types of ligand.

The possibility exists that EphA4 and EphB2 have some special relationship to the ancestor of vertebrate Eph receptors, given that these two receptors are uniquely capable of interacting with both ephrin-As and ephrin-Bs [3,4]. Indeed, the most common vertebrate matches for urochordate Eph receptors returned from BLAST searches are EphA4 and EphB2. EphA4 and EphB2 may therefore represent the most ancient vertebrate Eph receptors. By this scenario, the descendants of the ancestral Eph, which was presumably capable of interacting with both types of ligand, became increasingly specific for either ephrin-As or ephrin-Bs, such that EphA4 and EphB2 persist as the only reminders of the ancestral characteristic of dual ligand binding specificity. Our findings, however, fail to support this idea since neither EphA4 nor EphB2 is placed outside of a subclade containing the remaining EphA or EphB receptors. This suggests that vertebrate Eph receptors randomly lost the ability to interact with both ephrin-As and ephrin-Bs as they diversified, which is consistent with the fact that EphA4 cannot bind all ephrin-Bs just as EphB2 cannot bind all ephrin-As. However, the homology of the urochordate Eph receptors to EphA4 and EphB2 remains as a point of support for the idea that the predecessor of vertebrate Eph receptors could bind to both ephrin-As and ephrin-Bs.

Is there evidence that the diversification of Eph receptors in the vertebrates is associated with a diversification of function? The functions performed by Eph receptors and ephrins typically fall into one of several categories including regulation of axon guidance (e.g. formation of visual topographic maps), directed cell migration (e.g. NC cell pathfinding), or tissue segregation (e.g. hindbrain segmentation). Do EphA receptors perform different functions from EphB receptors? A survey of the literature reveals that while some specific roles are unique to each class of receptor, in a broader context both EphAs and EphBs perform a wide range of similar functions. For example, EphAs are uniquely involved in tissue segregation events that are part of somite segmentation [15,16] and limb development [17-20], as well as motor neuron axon guidance [21-27]. On the other hand, both EphAs and EphBs contribute to hindbrain segmentation [7,28-31], segregation of veins from arteries during angiogenesis [32-35], patterning of the inner ear [36,37], directed migration of NC cells [8,9,38,39], and guidance of RGC axons to their targets [39-41]. Thus, it is difficult to ascribe one set of functions to EphAs and another to EphBs. The impressive array of EphAs and EphBs may simply reflect the duplication and diversification of an ancestral receptor to perform subtle variations of the same functions in different cell or tissue types as the body plan become progressively more complex in the lineage from basal chordate to vertebrate.

Another intriguing question is if one of the three prototypical functions performed by Eph receptors can be regarded as ancestral. The surprising discovery of a poriferan Eph [10] indicates that these receptors are not a eumetazoan novelty. Sponges lack nerves, muscles, and true tissues, but do possess a cellular grade of organization in which different cell types are arranged into tissue-like layers such as the porous epidermis (pinacoderm) on the exterior and the lining of flagellated cells on the interior [42,43]. While there has been no research on Eph function in sponges, they may play a role in the positioning of these different cell types during development [44]. In the nematode *C. elegans*, Eph receptors and ephrins are expressed in adjacent populations of neural precursor cells and when either receptor or ligand function is disrupted, the neural precursors become disorganized and the ventral epidermis fails to close over these cells [45,56]. While the Eph receptors and ephrins are expressed in neural cells, the function performed here is more consistent with a tissue segregation mechanism. On the other hand, defective Eph function in *C. elegans *also results in axons extending beyond their expected target sites [47], indicating that these receptors can contribute to axon guidance in invertebrates as well. In the insect *D. melanogaster*, Eph receptors and ephrins function similarly to guide cortical axons to the optic ganglion [48] and restrict interneuronal axons from either crossing the midline or exiting the central nervous system [49]. In the echinoderm *S. purpuratus*, Eph receptors and ephrins are expressed in non-overlapping ectoderm domains and do not appear to function directly in axon guidance, but rather are involved in patterning domains of ectoderm (Jones, Yaguchi, and Burke, unpublished). In *C. intestinalis *embryos, Eph and ephrin mediate an early cell fate specification by activation of ERK signalling pathway producing an asymmetric cell division [50]. [51] has speculated that the ancestral function is regulation of cellular movements, but we currently lack sufficient functional data from diverse metazoans to reasonably conclude which functions of Eph receptors can be regarded as ancestral. A better understanding of the interacting signal transduction components of Eph and ephrin signalling pathways may reveal relationships not apparent from considerations of overall function.

A second important implication from our findings is that ephrin-Bs are the more ancient of the two types of vertebrate ephrins. The urochordate and vertebrate ephrin-Bs are actually part of a larger clade that also includes cnidarian, nematode, insect, echinoderm, and cephalochordate ephrins. The urochordate and vertebrate subclade is grouped with one comprised of cephalochordate, echinoderm, and cnidarian ephrins, while the nematode and insect ephrins form an external ecdysozoan clade. The four *C. elegans *ephrins are paraphyletic with the single *D. melanogaster *ephrin, perhaps indicating that the common ancestor for nematodes and insects possessed multiple ephrins, some of which were lost in the lineage to *D. melanogaster*. Although lineage specific expansion of ephrins occur, so an expansion of the C. elegans complement is equally likely.

Our analysis of Eph receptors suggests urochordate Eph receptors are more closely related to vertebrate receptors than the cephalochordate receptors, BfEph1 and BfEph2. Similarly, our analysis of ephrins allies vertebrate A-type ligands with urochordate A-type ligands. These topologies are contrary to the more firmly established relationship of cephalochordates being considered the closest living relatives of vertebrates. Similar results have been reported with other analyses involving small sets of molecules, yet more complete sets confirm the conventional relationships [12,52]. The evolutionary history of Eph receptors and ephrin ligands appears to be distinct from the history of the species, which suggests that functional features of these molecules have constrained their diversification.

The unusual topology of the ephrin-B clade suggests that the ligands possessed by cnidarians, echinoderms, and cephalochordates are more ephrin-B-like than those possessed by ecdysozoans. On the other hand the bootstrap support for the cnidarian, echindoderm, and cephalochordate ephrins grouping with the urochordate and vertebrate ephrin-Bs is low. Therefore, the possibility of a more obvious non-chordate plus cephalochordate subclade existing cannot be excluded. Although there is currently a wealth of genomic information available for an array of invertebrates, there are no Eph receptor or ephrin sequences from lophotrochozoans. This puts a considerable gap into our analysis, the elimination of which might allow for a more clear resolution of ephrin-B phylogeny.

Our analysis suggests that a clade of ephrin-A ligands distinguished by sequence similarity in the ephrin domain arose in a common ancestor of urochordates and vertebrates. Curiously, some of the invertebrate ephrins lack a transmembrane domain and have a putative GPI anchor site, which is the defining feature of the vertebrate A type ephrins. These invertebrate ephrins have an ephrin-B-like receptor binding domain that is the tethered to the cell membrane by a GPI anchor. Predictions of transmembrane domains occur in cnidarians, insects, urochordates, cephalochordates, and vertebrates. Whereas, GPI anchors predictions occur in nematodes, echinoderms, urochordates and vertebrates. This distribution suggests that a transmembrane anchorage of an ephrin with a B-type ligand binding domain is the ancestral state and that GPI anchors have arisen or been lost at least 3 times. The types of membrane anchorage have yet to be confirmed experimentally, but it suggests that the vertebrate ephrin As are a homologous group related by their binding domain and that the mechanism of anchorage is not a reliable defining feature for the group.

## Conclusion

We have shown through a phylogenetic analysis that Eph receptors and ephrin ligands followed different paths of evolution. For the receptors, the ancestral Eph receptors of non-chordates and cephalochordates diversified independently in urochordates and vertebrates. For the ligands there were lineage specific diversifications of forms with ephrin-B-like receptor binding domains and ephrin-As appear in the founding lineages of urochordates and vertebrates. Ephrins and Eph receptors therefore diversified at different points in evolution, such that the common ancestor of urochordates and vertebrates possessed a single receptor, but two ligands. Additional data on the functions and signalling pathways employed by Eph receptors and ephrins in diverse metazoans are necessary to more clearly define the evolutionary relationships within this intriguing family of signalling molecules.

## Methods

Full length Eph and/or ephrin amino acid sequences for *Ephydatia fluviatilis *(poriferan), *Nematostella vectensis *(cnidarian), *Caenorhabditis elegans *(nematode), *Drosophila melanogaster *(insect), *Strongylocentrotus purpuratus *(echinoderm), Branchiostoma floridae (cephalochordate), *Ciona intestinalis *(urochordate), and *Homo sapiens *(vertebrate) were retrieved from (53–55, see additional files 1, 2). Sequences were aligned in MEGA 4.0 (56) and assembled into phylogenetic trees using the minimum evolution (ME), maximum parsimony (MP), neighbour joining (NJ), and UPGMA methods. Sequences aligned in BioEdit (57) were assembled into maximum likelihood (ML) trees using the Treefinder (58) program. Trees were created under the Jones-Taylor-Thornton model of amino acid substitution (where appropriate) using 1000 bootstrap replications. In each case, the ME, ML, and NJ trees gave very similar results and so we have included only the ML trees here. GPI modification site predictions were done at (59) and transmembrane domain predictions were made at the TMHMM server (60) (see Additional file 3).

## Authors' contributions

DOM did the analysis and prepared the first draft of the paper; RDB conceived of the study, revised, and edited the manuscript. All authors read and approved the final manuscript.

## Supplementary Material

Additional file 1 Aligned Eph sequences. Aligned Eph amino acid sequence data used in this study. Click here for file

Additional file 2 Aligned Ephrin sequences. Aligned Ephrin amino acid sequence data used in this study. Click here for file

Additional file 3 Accession numbers. A list of accession number for the amino acid sequences used in this study. Click here for file
